# Immune Phenotypes in Patients With Invasive Mould Infection Support the Use of PD‐1 Inhibition as Potential Treatment Option

**DOI:** 10.1111/myc.70044

**Published:** 2025-03-17

**Authors:** Sibylle C. Mellinghoff, Martin Thelen, Michael von Bergwelt‐Baildon, Hans A. Schlößer, Oliver A. Cornely, Rosanne Sprute, Jannik Stemler, Leonie Mayer, Leonie Marie Weskamm, Monika Friedrich, My Linh Ly, Christine Dahlke, Marylyn M. Addo

**Affiliations:** ^1^ Department I of Internal Medicine, Center for Integrated Oncology Aachen Bonn Cologne Duesseldorf (CIO ABCD) and Excellence Center for Medical Mycology (ECMM), Faculty of Medicine and University Hospital Cologne University of Cologne Cologne Germany; ^2^ German Centre for Infection Research (DZIF), Partner Site Bonn‐Cologne Cologne Germany; ^3^ Institute of Translational Research, Cologne Excellence Cluster on Cellular Stress Responses in Aging‐Associated Diseases (CECAD), Faculty of Medicine and University Hospital Cologne University of Cologne Cologne Germany; ^4^ Center for Molecular Medicine Cologne, Faculty of Medicine and University Hospital Cologne University of Cologne Cologne Germany; ^5^ Department of General, Visceral, Thoracic, and Transplantation Surgery, Faculty of Medicine and University Hospital Cologne University of Cologne Cologne Germany; ^6^ Department III of Internal Medicine Ludwig Maximilian University of Munich Munich Germany; ^7^ German Cancer Consortium (DKTK) Munich Germany; ^8^ Comprehensive Cancer Center München‐LMU (CCCMLMU) LMU Munich Munich Germany; ^9^ Clinical Trials Centre Cologne (ZKS Köln), Faculty of Medicine and University Hospital Cologne University of Cologne Cologne Germany; ^10^ Department of Clinical Immunology of Infectious Diseases Bernhard Nocht Institute for Tropical Medicine Hamburg Germany; ^11^ Institute for Infection Research and Vaccine Development (IIRVD) University Medical Centre Hamburg‐Eppendorf Hamburg Germany; ^12^ German Centre for Infection Research (DZIF), Partner Site Hamburg‐Lübeck‐Borstel‐Riems Hamburg Germany

**Keywords:** aspergillosis, immune checkpoint, mucormycosis, PD‐1

## Abstract

**Background:**

Invasive mould infections (IMI) cause substantial morbidity and mortality in populations at risk. Novel treatment approaches are urgently needed. Targeting immune checkpoints may reverse hyporesponsiveness of the innate and adaptive immune systems.

**Methods:**

In this prospective, observational study, we investigated immune checkpoint expression levels on immune cells in patients with invasive aspergillosis (IA; *n* = 25) and mucormycosis (MU; *n* = 7). Healthy controls (HC; *n* = 5) and patients with matched haematological diseases but without IMI served as control populations (CP; *n* = 10). Multicolour flow cytometry analysis was used to compare immune cell subsets and the expression of immune‐regulatory molecules in peripheral blood mononuclear cells (PBMCs).

**Results:**

Lymphocyte subsets and immune phenotypes in PBMCs were similar between patients with IMI and haematological CP, except for regulatory T cells, which were increased in PBMCs of patients with IA and MU compared to HCs. In IA and MU, PBMCs showed increased expression of immune checkpoint molecules compared to healthy controls and matched haematological CP, with this effect being more pronounced in IA than in MU. We found heterogeneous, disease‐, molecule‐, and patient‐specific expression patterns of immune checkpoint molecules. For example, PD‐1 expression was highest in MU PBMCs, followed by IA PBMCs, while HC PBMCs showed lower expression levels. Overall mortality in our patient population was 44.0% (IPA) and 80.0% (MU).

**Conclusions:**

We report an immune phenotype consistent with T‐cell exhaustion in IMI, indicating potential contributions from haematological treatment, underlying disease, and infection. However, the primary underlying cause remains unclear and requires further investigation. A marker that was notably higher in IMI patients was PD‐1, and treatment approaches specifically targeting this molecule may be promising.

## 1 Introduction

Invasive mould infections (IMI) by *Aspergillus* spp. and Mucorales cause substantial morbidity and mortality in populations at risk even despite treatment with antifungal agents [1, 2, 3]. Most cases of invasive aspergillosis (IA) are caused by *Aspergillus fumigatus* , *A. flavus* , *A. niger* , and *A. terreus* in decreasing order, albeit their prevalence may vary [4, 5, 6]. Invasive mucormycosis (MU) is caused by a variety of fungi from the order Mucorales, with *Rhizopus* and *Mucor* spp. being most commonly encountered clinically. Risk factors encompass haematological malignancies with disease or treatment‐associated neutropenia and respiratory infections by viruses such as influenza or SARS‐CoV‐2 [7, 8]. For IA, mortality can exceed 26% [1, 9, 10, 11] even if treated adequately. The outcome of patients with MU is even more devastating; a mortality rate of 62% is reported despite antifungal treatment [12]. Management of these IMI remains challenging to clinicians in spite of available guidelines and antifungal stewardship initiatives [2, 3].

Immunocompromised patients face the highest risk for IMI, supporting the hypothesis that strengthening host immunity may improve outcomes. Preclinical studies [13, 14, 15, 16, 17, 18, 19] in murine models of invasive fungal infections (IFI), including cryptococcal and paracoccidioidomycosis, Aspergillus, and Mucormycosis infections, have demonstrated that enhancing immune responses through interventions such as immune checkpoint blockade, IL‐7, or IFN‐γ can significantly improve fungal clearance and survival. These models show that therapies targeting immune dysfunction not only enhance fungicidal activity but also promote pathogen clearance from infected tissues. Limited human data further support the potential of such immunotherapeutic strategies as adjunctive treatments to improve outcomes in this highly vulnerable population [20]. Additionally, recent research indicates a potential benefit of immune checkpoint inhibition (ICI) in managing IMI [14, 15, 16, 21, 22]. Immune activation is delicately regulated by positive and negative co‐stimulatory molecules to maintain a balance. Inhibitory immune checkpoints modulate immune activation to ensure self‐tolerance [23, 24, 25, 26].

The programmed cell death 1 (PD‐1) molecule, expressed on lymphocytes, monocytes, and natural killer (NK) cells, is crucial in this regulation [27]. Its ligands, PD‐L1 and PD‐L2, are expressed on antigen‐presenting cells, while PD‐L1 is found on various non‐haematopoietic cells, including tumour cells. Binding of PD‐1 to its ligands results in the inhibition of T cell receptor signalling, dampening T cell functions. Inhibiting the PD‐1/PD‐L1 axis has shown promise in restoring immune function and is successfully employed in cancer treatment [28, 29, 30, 31, 32, 33, 34]. Other immune checkpoint molecules such as LAG3, Tim3, and CTLA4 also critically impact the regulation of T cell function and immune homeostasis. LAG3 (lymphocyte‐activation gene 3) is involved in dampening T cell activity and is often co‐expressed with PD‐1 during chronic infection, contributing to T cell exhaustion. Tim3 (T‐cell immunoglobulin and mucin‐domain containing‐3) negatively regulates immune responses by interacting with ligands like galectin‐9, further impairing T cell functionality in the setting of persistent infections [35]. CTLA4 (cytotoxic T‐lymphocyte‐associated protein 4) competes with CD28 for binding to costimulatory molecules, reducing T cell activation [36].

Targeting immune checkpoints may reverse hyporesponsiveness of innate and adaptive immunity during IMI, potentially supporting the immune response. Several reports have documented successful adjunctive therapy with ICI in patients with IMI [21, 37, 38, 39, 40]. Notably, increased PD‐1 expression on T cells has been observed in patients with *Candida* bloodstream infections [22] highlighting the potential relevance of immune checkpoint expression in IFI. Data on immune checkpoint expression in patients with IMI are missing to date.

In this study, we investigate immune checkpoint expression levels on immune cells of patients with IA or MU.

## 2 Materials and Methods

### 2.1 Study Design and Setting

This bi‐centric prospective observational cohort study was performed at the University Hospital of Cologne and at the University Medical Centre Hamburg‐Eppendorf. Patients were included consecutively from 04/2017 to 10/2020.

### 2.2 Patients and Samples

Patients with proven or probable IA (*n* = 25) and MU (*n* = 7) were included. IMI was defined by the revised EORTC/MSG criteria from 2020 [41]. Sample collection was performed as soon as possible after the diagnosis of IMI. On average, they were assessed 4 days after the diagnosis of IMI (4.4, range 1 to 18 days). Peripheral blood mononuclear cells (PBMCs) of healthy controls (*n* = 5) as well as PBMCs obtained from patients with matched haematological diseases and without IMI (control patients: CP; *n* = 10) served as control populations. Written informed consent was signed by all patients, and this study was approved by the institutional ethics committee Cologne (No. 20‐1368_1) and Hamburg (No. PV4780). This study was registered at clinicaltrials.gov (NCT04533087).

### 2.3 Data Assessment

Patient characteristics and details on IMI (day of diagnosis, treatment, response, or progress) were assessed. We collected data on outcomes (survival at day 30, 120) and, in case of death, attribution to IMI upon the judgement of the treating physician. Clinical data were accessible to all participating researchers, including the performers and readers of the immunological assays.

### 2.4 Preparation of PBMCs

Blood was collected in EDTA S‐Monovette tubes (Sarstedt, Germany). PBMCs were isolated using density‐based separation with Histopaque1077 (Sigma‐Aldrich, Germany). A maximum of 5–10 × 10^6^ PBMCs per 2.0 mL CryoPure tube (Sarstedt, Germany) was resuspended in fetal bovine serum (FBS) supplemented with 10% dimethyl sulfoxide and immediately transferred to a controlled‐rate freezing container (Stratacooler, Agilent, USA) and stored overnight at −80°C. Frozen vials were then transferred to liquid nitrogen until analysis.

### 2.5 Flow Cytometric Phenotyping of Lymphocytes

PBMCs were thawed using RPMI medium supplemented with 20% FBS. 2 × 10^5^ PBMCs per well were stained in a 96‐well round‐bottom plate (BRAND, Germany) for flow cytometry (detailed antibody list in Table S1 and detailed gating strategy in Figure S1) and acquired on an LX Cytoflex flow cytometer (Beckman Coulter, Germany). Live‐dead staining was performed for 15 min at 4°C. Cells were washed using CellWASH (BD, USA). Cells were resuspended in a master mix containing antibodies of interest and incubated for 20 min at 4°C. FoxP3 staining was performed using the Foxp3/Transcription Factor Staining Buffer Set (Invitrogen, USA) according to the manufacturer's instructions adjusted to a 96‐well round‐bottom plate. All washing steps were performed at 500 × g. Data were analysed using Kaluza software v.2.1 (Beckman Coulter, USA). Samples containing < 100 CD45^+^ cells were excluded from further analysis.

### 2.6 Statistical Analyses and Visualisation

Statistical parameters and used tests are included in the respective figure legends. Applicable statistical tests were performed using GraphPad v8.3.0 (GraphPad Prism, USA) as indicated in the figure legends. K‐means clustering and principal component analysis were performed using Orange Data Mining v3.28. Representative FACS plots were exported from Kaluza v2.1 (Beckman Coulter, USA) and graphs were generated using GraphPad v8.3.0. Figures were created using Inkscape v1.0beta1.

## 3 Results

### 3.1 Patient Characteristics

Patient characteristics of patients with IMI are given in Table S2. Mean age was 53 years (range 15 to 79 years). The most frequent risk factor for IMI was underlying haematological malignancy. Twenty‐one patients were neutropenic at the onset of infection (17 with IA and 4 with MU). In 6 of 25 patients, IA was proven; 19 had probable IA. All patients with MU had proven infection.

### 3.2 Lymphocyte Subsets and Immune Phenotypes in PBMCs Are Similar Between Patients With IMI and Haematological Control Patients

**FIGURE 1:**
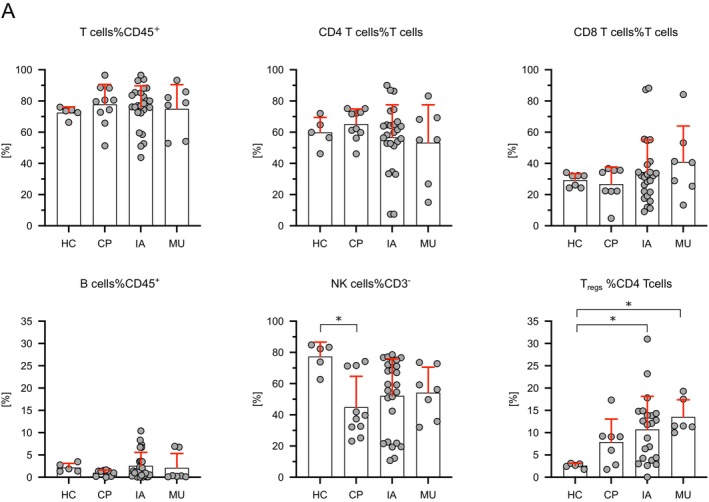
Comparable composition of lymphocyte subsets in PBMCs of healthy individuals, cancer patients, and patients with invasive aspergillosis or mucormycosis. The lymphocyte composition in PBMCs from healthy controls (HC, *n* = 5), cancer patients (CP, *n* = 10), and patients with invasive aspergillosis (IA, *n* = 25) or mucormycosis (MU, *n* = 7) was analysed by flow cytometry. Samples containing fewer than 100 viable lymphocytes (CD45^+^ cells) were excluded (a detailed gating strategy is provided in Figure S1). The percentage for each sample is shown with the mean (bar) ± standard deviation (red). Significant differences between all groups were calculated using a nonparametric Kruskal‐Wallis test followed by Dunn’s post hoc test and are indicated by asterisks (*p ≤ 0.05).

We used multicolour flow cytometry analysis to compare immune cell subsets and expression of immune‐regulatory molecules in PBMCs from healthy controls (HC PBMCs) and PBMCs from patients with IMI (IA/MU PBMCs). The majority of patients with IA (21/25) and all patients with MU (7/7) had an underlying haematological disease. We therefore analysed PBMCs from a cohort of haematological control patients (CP PBMCs) without IMI. As depicted in Figure 1, similar percentages of B lymphocyte and T lymphocyte subsets were observed in PBMCs of IA when compared to HCs. We saw lower percentages of NK cells in CP compared to HCs, but no significant difference when compared to patients with IMI. Patients with IA and MU showed increased percentages of regulatory T cells (Tregs) compared to HCs (Figure 1; *p* = 0.0289 and *p* = 0.0137).

### 3.3 PBMCs of Patients With IMI Show Increased Expression of Immune Checkpoint Molecules

**FIGURE 2:**
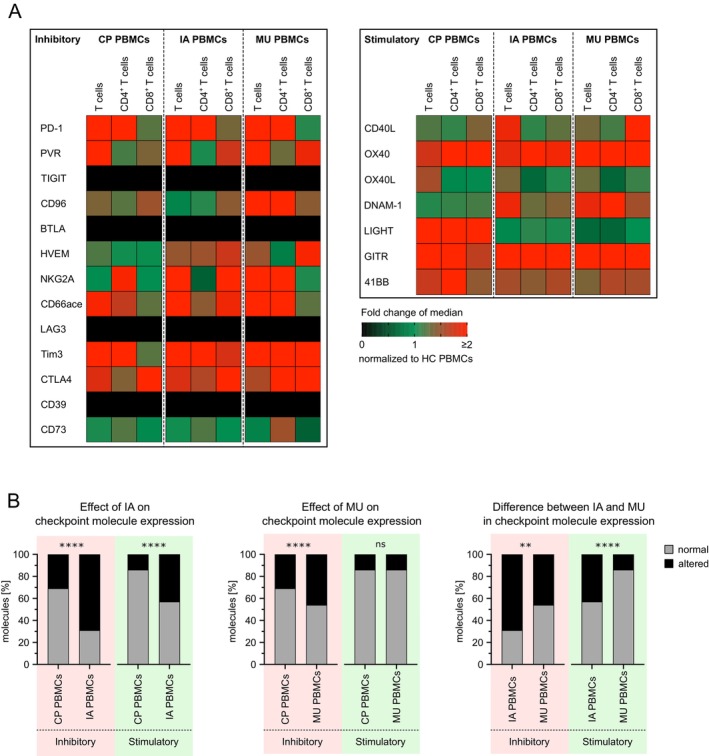
Cancer and invasive infection facilitate altered expression of immune checkpoint molecules in PBMCs. The expression of immune checkpoint molecules on T cells, CD4^+^ T cells, and CD8^+^ T cells in PBMCs from healthy controls (HC, *n* = 5), cancer patients (CP, *n* = 10), and patients with invasive aspergillosis (IA, *n* = 25) or mucormycosis (MU, *n* = 7) was analysed by flow cytometry. Samples containing fewer than 100 viable lymphocytes (CD45^+^ cells) were excluded (a detailed gating strategy is provided in Figure S1). (A) The median‐to‐median fold change in immune checkpoint molecule expression was calculated between healthy individuals and cancer patients, as well as between healthy individuals and patients with invasive aspergillosis or mucormycosis. (B) Significant differences in immune checkpoint molecule expression between healthy individuals (*n* = 5) and patients with cancer, invasive aspergillosis, or invasive mucormycosis were calculated using a nonparametric, two‐tailed Mann–Whitney test. The percentage of altered immune checkpoint molecules (significant upregulated or downregulated) was compared between CP and IA or MU to determine the effects of invasive infection and cancer on immune checkpoint molecule expression. Additionally, the percentage of altered molecules in IA and MU was compared to assess the effect of different invasive infections on immune checkpoint molecule expression.

Fractions of T cells expressing 13 co‐inhibitory and 7 co‐stimulatory molecules in IMI (IA/MU) PBMCs were compared to the respective control populations. A median‐to‐median fold change in immune checkpoint molecule expression was calculated for CP, IA, and MU relative to HCs. Compared to HCs, PBMCs of patients with IA and MU generally showed increased expression of immune checkpoint molecules on CD3^+^ T cells, most of which were co‐inhibitory. A summary of this median‐to‐median analysis is provided in Figure 2A. Some molecules, such as PD‐1, displayed a similar pattern in CP, IA, and MU PBMCs, while others, such as CD96, showed a stronger alteration in MU PBMCs but not in CP or IA PBMCs. Similar observations were made for co‐stimulatory molecules. For example, OX40 was increased in CP, IA, and MU PBMCs, whereas LIGHT was only increased in CP PBMCs (Figure 2A). CD4^+^ T cells and CD8^+^ T cells showed a balanced pattern of up‐ and down‐regulation of co‐inhibitory molecules. While PD‐1 showed a stronger increase on CD4^+^ T cells in CP, IA, and MU PBMCs, PVR was increased on CD8^+^ T cells but not on CD4^+^ T cells.

Next, we analysed the number of significantly altered immune checkpoint molecules in CP, IA, and MU compared to HCs (Figure S2). By comparing IA and MU PBMCs to CP PBMCs, we evaluated the impact of IMI on checkpoint expression, finding a stronger effect in IA than in MU (Figure 2B). Of note, IA PBMCs showed a significantly higher number of altered co‐inhibitory as well as co‐stimulatory molecules compared to CP PBMCs (Figure 2B,p < 0.0001). In contrast, a lower number of significantly altered co‐inhibitory molecules, and no additional effect of the infection on the expression of co‐stimulatory molecules was detected in MU PBMCs (Figure 2B).

**FIGURE 3:**
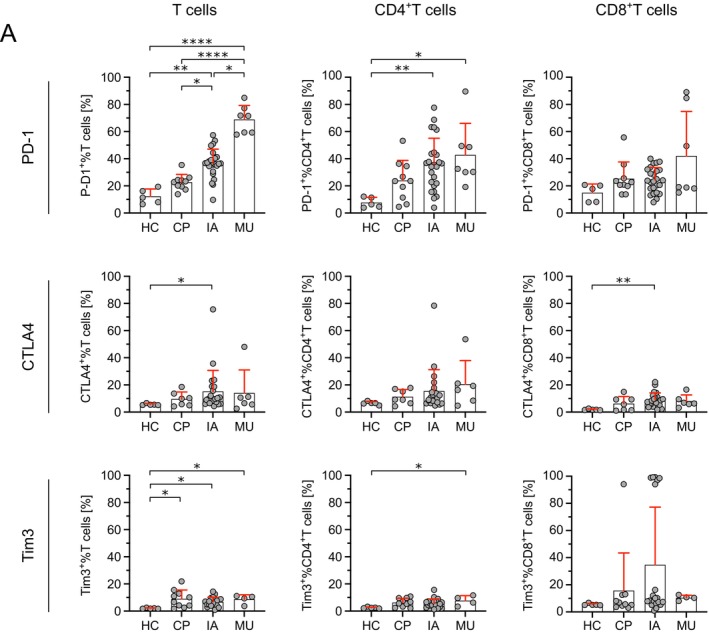
Altered expression of immune checkpoint molecules in patients with cancer and invasive infections. The expression of immune checkpoint molecules on T cells, CD4^+^ T cells, and CD8^+^ T cells in PBMCs from healthy controls (HC, *n* = 5), cancer patients (CP, *n* = 10), and patients with invasive aspergillosis (IA, *n* = 25) or mucormycosis (MU, *n* = 7) was analysed by flow cytometry. Samples containing fewer than 100 viable lymphocytes (CD45^+^ cells) were excluded (a detailed gating strategy is provided in Figure S1). Individual percentages are depicted with mean (bar) ± standard deviation (red). Significant differences were calculated using a nonparametric Kruskal‐Wallis test followed by Dunn's post hoc test and are indicated by asterisks (*p ≤ 0.05, **p ≤ 0.01, ****p ≤ 0.0001).

T cells expressing co‐inhibitory molecules were particularly increased in IA PBMC. Among others, expression was increased in IA PBMCs for PD‐1, CTLA4, and Tim‐3. PD‐1 expression was highest in MU PBMCs (68.8% ± 10.4), followed by IA PBMCs (36.6% ± 10.5), whereas HC PBMCs showed lower expression levels (12.3% ± 5.4). Expression patterns varied by disease, patient, and molecule, with IA showing significant PD‐1 upregulation in CD4^+^ T cells (35.9% ± 19.3, *p* = 0.0076). Detailed data are in Figure 3 and Figures [Link], [Link]. In summary, the observed additive alteration in the expression of immune‐regulatory molecules in patients with IMI, compared to those with haematologic disease but without IMI, suggests a systemic exhausted T‐cell phenotype driven by the infection, with a highly heterogenous expression pattern across patients and molecules.

### 3.4 Outcome

Overall d30 mortality in our patient population was 44.0% (IA) and 80.0% (MU). Mean time to death was 32.9 days. Seven patients (46.7%) died within 1 week from diagnosis. The treating physicians attributed 7/11 deaths to IPA and 4/4 to MU. Mortality at day 30 was 40.0% and at day 90 44.0% in patients with IA, 80.0% at day 30 as well as day 90 in patients with MU.

## 4 Discussion

Innate and adaptive immune systems seem to be impaired during severe IFI [13, 42, 43, 44, 45]. Both in vitro and in vivo studies imply that strengthening host immunity could possibly improve outcomes in IFI such as candidiasis [13, 16, 20, 21, 22, 46]. By analysing lymphocyte immune phenotypes of 30 patients with IMI and controls, we comprehensively depict patterns of T cell exhaustion in IMI.

Distribution of typical risk factors for IMI was in line with previously published data [1]. The mortality in our study (46.7%) was comparable to previous reports [11, 47] and was attributable to IFI in 73.3%. Most patients died within the first 3 weeks of infection.

Our results from patients with IMI display an immune phenotype consistent with T cell exhaustion. These data are in line with the only previously published data from two patients with IMI (e.g., up‐regulation of TIM3 and CTLA4) [37, 38]. T cell exhaustion implies a poorly functional immune response with reduced cytokine production, decreased proliferation, and premature apoptotic cell death. Increased expression of the inhibitory immune checkpoint receptor PD‐1 and its ligands, but also further receptors such as TIM3, LAG3, CTLA4, BTLA, or TIGIT, mediate T cell hypo‐responsiveness. Our data indicate a high prevalence of PD‐1^+^ T cells, especially in patients with invasive mucormycosis (MU). Notably, this upregulation of PD‐1 is not mirrored in both CD4^+^ and CD8^+^ T cell subsets, suggesting that the elevated PD‐1 expression may involve other, less well‐characterised T cell populations. Preclinical murine models have shown significant PD‐1 expression in double‐negative (DN) T cells, which lack both CD4 and CD8 markers. Although murine studies suggest PD‐1 expression in DN T cells [48, 49], human data on this population remain limited, and future studies should explore the phenotypic characteristics of these cells.

Furthermore, our analysis comparing immune checkpoint expression in PBMCs from IMI patients to controls (HCs and CP) demonstrated that IA had a more profound impact on immune checkpoint molecule expression. Specifically, IA was associated with a significantly higher number of altered co‐inhibitory and co‐stimulatory molecules compared to MU, with only a few distinctions between these groups, such as CTLA4 and BTLA. Notably, co‐stimulatory markers on CD3^+^ T cells were upregulated in IA but downregulated in MU, suggesting differing effects of infection on the immune profile in these conditions. T cells expressing co‐inhibitory molecules were most elevated in IA PBMCs, and similar upregulation was observed in non‐infected CPs with haematological malignancies. Among the significantly altered markers, PD‐1 and CD39 exhibited marked differences between IMI and CPs, with PD‐1 expression being highest in MU PBMCs, intermediate in IA, and lowest in healthy controls. Expression patterns varied according to disease, patient, and specific marker, with significant upregulation of PD‐1 observed in CD4^+^ T cells in IA. These findings indicate that although immune dysregulation is evident in both IA and MU, IA induces a more pronounced alteration in immune checkpoint expression, suggesting that IA infection may exert a greater impact on T cell function. In summary, our data highlight that IMI contributes to a cumulative alteration in immune‐regulatory molecule expression that extends beyond the effects of underlying haematological malignancies. The upregulation of key co‐inhibitory markers such as PD‐1 and CTLA4 in IA and MU suggests a functional impairment of T cells, likely contributing to compromised antifungal immunity. These observations underscore the potential role of immune exhaustion in the pathophysiology of IMI. Given the widespread alteration of immune checkpoints in these patients, further research is essential to evaluate the functional status of these exhausted T cells and to explore therapeutic strategies targeting immune checkpoints, which could help restore effective immune responses in IMI.

We saw lower percentages of NK cells in IA compared to HC, but not compared to CP with haematological malignancies. The antifungal activity of NK cells in IFI is mediated through direct damage to the fungus by cytotoxic molecules and indirectly via the release of cytokines and subsequent immune activation [50]. In patients after allogeneic haematopoietic stem cell transplantation, a higher absolute NK cell count was associated with higher cure rates [51]. In another study, a low NK count in solid organ transplant patients independently predicted IMI [52]. While our data suggest this trend as well, further research should take NK cells into account when analysing exhaustion phenotypes in the context of IMI. In addition, the role of maturation markers on mononuclear phagocytes, such as MHC II expression on monocytes and macrophages, seems to be important. They are reported to be critical indicators of immune function and have been highlighted in both IFI and the sepsis literature [53, 54, 55, 56]. Maturation of these cells plays a significant role in immune responses, and alterations in their expression may contribute to immune dysfunction during infection. Thus, evaluating the expression of these maturation markers in future studies would provide important insights into the mechanisms underlying antifungal immunity and potential immune paralysis in the context of IMI.

Due to a comparable immune phenotype in matched control patients, we assume that T cell exhaustion may as well be rather associated with treatment and disease than with infection. However, the only marker that is relevantly higher in IMI patients is PD‐1, and a treatment approach targeting specifically this molecule is tempting. Of note, immunologic phenotypes are very similar in patients with IA and IM. In contrast to candidemia, where T cell exhaustion was shown to be an effect of the infection itself [22, 57], this is not surprising considering IMI as localised tissue‐invasive infections. Future studies should include samples to evaluate the local environment at the infection site, for example, tissue and bronchoalveolar lavage (BAL) samples, providing insights into tissue‐specific immune responses that may inform more targeted treatments for IMI. While especially MU tends to disseminate quickly, a second time point of observation would be highly interesting. However, data from patients treated with ICI in this setting imply a benefit likely attributable to systemic immune enhancement during actively ongoing infection [21, 37, 38, 39, 40].

Numerous critical factors that could impede the translation of observed phenomena into clinical benefits for patients remain to be fully understood. For instance, the optimal timing for initiating treatment must be meticulously determined. Here, we studied the immune phenotype a few days after the diagnosis of IMI. Immune exhaustion, including checkpoint induction, is a dynamic process starting within days after IMI; therefore, taking antifungal treatment into account, an early assessment may not encompass its full manifestation but rather give an idea of a commencing process. The study's progress was hindered by early deaths, resulting in a limited number of patients available for follow‐up. However, future studies may include a larger cohort with extended follow‐up periods to better comprehend treatment effects across different infection phases and potential co‐infections, which may exhibit varying susceptibility to ICI. Diverse investigational agents targeting immune modulation have been explored in the management of other infectious diseases, with potential efficacy observed in the early phase of severe infections [58, 59, 60]. However, caution is warranted as they may prove detrimental if administered during the subsequent immunosuppressive phase. Therefore, clinical trials investigating ICI in IMI should incorporate sequential immune phenotyping and functional assays to delineate longitudinal immune changes, thereby facilitating tailored treatment strategies.

This study has some important limitations. Samples were collected at differing time points, owing to the setting of diagnosis and management in these patients; in addition, we were unable to perform longitudinal observations. The group of 30 patients was subdivided by IMI pathogen and heterogeneous underlying diseases, and the small control cohorts may not allow us to generalise our findings. Moreover, neutropenic patients with dysfunctional lymphocytes may be less responsive to checkpoint inhibition. Yet, lymphocytopenia is often incomplete, and many patients still have functional lymphocytes, particularly T cells, during neutropenia. This indicates a possible role also in this subpopulation. While our study investigated IMI only, our findings may also help understand other systemic infections. Exploring potential associations between checkpoint expression or its reversal and clinical outcomes, such as response to antifungal therapy or survival, would be a valuable direction for future research. However, the heterogeneity of our cohort and the limited sample size underscore the need for larger, multicenter studies to better assess these relationships and identify predictive biomarkers for therapeutic success in IMI.

In conclusion, we report an immune phenotype consistent with T cell exhaustion in IMI, which appears to be an effect of the predisposing malignancy, its treatment, and the fungal infection. However, the only marker substantially increased in IMI patients is PD‐1, thus supporting a treatment approach targeting specifically this molecule.

## Author Contributions

**Sibylle C. Mellinghoff:** conceptualization, investigation, methodology, validation, visualization, funding acquisition, writing – original draft, resources, project administration. **Martin Thelen:** conceptualization, methodology, validation, formal analysis, visualization, writing – original draft. **Michael von Bergwelt‐Baildon:** conceptualization, writing – review and editing. **Hans A. Schlößer:** conceptualization, writing – review and editing, methodology. **Oliver A. Cornely:** conceptualization, writing – review and editing, methodology, funding acquisition. **Rosanne Sprute:** conceptualization, writing – review and editing. **Jannik Stemler:** conceptualization, writing – review and editing. **Leonie Mayer:** methodology, writing – review and editing. **Leonie Marie Weskamm:** methodology, writing – review and editing. **Monika Friedrich:** methodology, writing – review and editing. **My Linh Ly:** data curation, validation, methodology. **Christine Dahlke:** methodology, validation, writing – review and editing. **Marylyn M. Addo:** conceptualization, methodology, writing – review and editing.

## Conflicts of Interest

S.C.M. reports grants from DZIF (Clinical Leave Stipend and Advanced Clinician Scientist Stipend) as well as honoraria from Pfizer. O.A.C. reports grants or contracts from BMBF, Cidara, DZIF, EU‐DG RTD, F2G, Gilead, MedPace, MSD, Mundipharma, Octapharma, Pfizer, Scynexis; Consulting fees from Abbvie, AiCuris, Basilea, Biocon, Boston Strategic Partners, Cidara, Seqirus, Gilead, GSK, IQVIA, Janssen, Matinas, MedPace, Menarini, Molecular Partners, MSG‐ERC, Mundipharma, Noxxon, Octapharma, Pardes, Partner Therapeutics, Pfizer, PSI, Scynexis, Seres, Shionogi, The Prime Meridian Group; Speaker and lecture honoraria from Abbott, Abbvie, Akademie für Infektionsmedizin, Al‐Jazeera Pharmaceuticals/Hikma, amedes, AstraZeneca, Deutscher Ärzteverlag, Gilead, GSK, Grupo Biotoscana/United Medical/Knight, Medscape/WebMD, MedUpdate, MSD, Moderna, Mundipharma, Noscendo, Paul‐Martini‐Stiftung, Pfizer, Sandoz, Seqirus, Shionogi, streamedup!, Touch Independent, Vitis; Payment for expert testimony Cidara; Participation on a DRC or DSMB for Cidara, IQVIA, Janssen, MedPace, PSI, Pulmocide. M.M.A. received support for research from the German Research Foundation (DFG), DZIF, BMBF, CEPI, and a lecture honorarium from Gilead (Campus Infectiology). O.A.C. reports grants or contracts from BMBF, Cidara, EU‐DG RTD (101037867), F2G, Gilead, MedPace, MSD, Mundipharma, Octapharma, Pfizer, Scynexis; Consulting fees from Abbvie, AiCuris, Biocon, Cidara, Gilead, IQVIA, Janssen, Matinas, MedPace, Menarini, Moderna, Molecular Partners, MSG‐ERC, Noxxon, Octapharma, Pfizer, PSI, Scynexis, Seres; Honoraria for lectures from Abbott, Abbvie, Al‐Jazeera Pharmaceuticals/Hikma, Gilead, Grupo Biotoscana/United Medical/Knight, MedScape, MedUpdate, Merck/MSD, Noscendo, Pfizer, Shionogi, streamedup!; Payment for expert testimony from Cidara; Participation on a Data Safety Monitoring Board or Advisory Board from Boston Strategic Partners, Cidara, IQVIA, Janssen, MedPace, PSI, Pulmocide, Shionogi, The Prime Meridian Group; A patent at the German Patent and Trade Mark Office (DE 102021 113007.7); Stocks from CoRe Consulting, EasyRadiology; Other interests from Wiley. Michael von Bergwelt‐Baildon (M.B.‐B.) received payment for consulting and appraisal work from AMGEN, MSD Sharp & Dohme, Novartis, Roche, KITE/Gilead, Bristol‐Myers Squibb, Astellas, Mologen and Miltenyi. He has personal fees from AMGEN, MSD Sharp & Dohme, Novartis, Roche, KITE/Gilead, Bristol‐Myers Squibb, Astellas, Mologen and Miltenyi. M.B.‐B. received funding for scientific research from AMGEN, MSD Sharp & Dohme, Novartis, Roche, KITE/Gilead, Bristol‐Myers Squibb, Astellas, Mologen and Miltenyi. M.B.‐B. has financial connections to AMGEN, MSD Sharp & Dohme, Novartis, Roche, KITE/Gilead, Bristol‐Myers Squibb, Astellas, Mologen and Miltenyi. Michael Bitzer (M.B.) received honoraria for consulting and appraisal work from Roche Pharma AG, Incyte Biosciences Germany GmbH, Bayer Vital GmbH, Bristol‐Myers Squibb GmbH & Co KgaA and MSD Sharp & Dome GmbH. H.A.S. discloses funding for research by AstraZeneca and Tabby Therapeutics and honoraria for advisory boards from BMS. The remaining authors declare no competing financial interests for this study. J.S. has received research support from the German Ministry of Education and Research (BMBF), Basilea Pharmaceuticals, Noscendo; has received speaker honoraria from AbbVie, Gilead, Hikma and Pfizer; and has been a consultant to Gilead, Alvea Vax and Micron Research all outside the submitted work.

## Supporting information


**Figure S1.** Gating strategy. Peripheral blood mononuclear cells were analysed by flow cytometry. Dead cells were excluded (alive). Lymphocytes were selected by gating for size and granularity (forward scatter (FCS‐A) and side scatter (SSC‐A)). Living lymphocytes were further gated for CD45^+^ lymphocytes (CD45^+^). B cells were defined by gating on CD19, whereas T cells were defined as CD3^+^ cells. T cells were then gated for their expression of CD4 and CD8. CD3 negative cells expressing CD56 were considered NK cells. A detailed list of antibodies, clones, and fluorochromes is provided in Table S1.

**Figure S2.** Significant alteration of immune checkpoint molecule expression in patient PBMCs. The expression of immune checkpoint molecules on T cells, CD4^+^ T cells, and CD8^+^ T cells in PBMCs from healthy controls (HC, *n* = 5), cancer patients (CP, *n* = 10), and patients with invasive aspergillosis (IA, *n* = 25) or mucormycosis (MU, *n* = 7) was analysed by flow cytometry. Samples containing fewer than 100 viable lymphocytes (CD45^+^ cells) were excluded (a detailed gating strategy is provided in Figure S1). Significant differences of immune checkpoint molecule expression between HCs and CP, IA, or MU PBMCs were calculated using a tow‐tailed, nonparametric Mann–Whitney test.

**Figure S3.** Co‐inhibitory molecule expression in patient PBMCs. The expression of co‐inhibitory molecules on T cells, CD4^+^ T cells, and CD8^+^ T cells in PBMCs from healthy controls (HC, *n* = 5), cancer patients (CP, *n* = 10), and patients with invasive aspergillosis (IA, *n* = 25) or mucormycosis (MU, *n* = 7) was analysed by flow cytometry. Samples containing fewer than 100 viable lymphocytes (CD45^+^ cells) were excluded (a detailed gating strategy is provided in Figure S1). Individual percentages are depicted with mean (bar) ± standard deviation (red). Significant differences between all groups were calculated using a nonparametric Kruskal‐Wallis test followed by Dunn’s post hoc test and are indicated by asterisks (*p ≤ 0.05, **p ≤ 0.01).

**Figure S4.** Co‐inhibitory molecule expression in patient PBMCs. The expression of co‐inhibitory molecules on T cells, CD4^+^ T cells, and CD8^+^ T cells in PBMCs from healthy controls (HC, *n* = 5), cancer patients (CP, *n* = 10), and patients with invasive aspergillosis (IA, *n* = 25) or mucormycosis (MU, *n* = 7) was analysed by flow cytometry. Samples containing fewer than 100 viable lymphocytes (CD45^+^ cells) were excluded (a detailed gating strategy is provided in Figure S1). Individual percentages are depicted with mean (bar) ± standard deviation (red). Significant differences between all groups were calculated using a nonparametric Kruskal‐Wallis test followed by Dunn’s post hoc test and are indicated by asterisks (*p ≤ 0.05, **p ≤ 0.01, ***p ≤ 0.001).

**Figure S5.** Co‐stimulatory molecule expression in patient PBMCs. The expression of co‐stimulatory molecules on T cells, CD4^+^ T cells, and CD8^+^ T cells in PBMCs from healthy controls (HC, *n* = 5), cancer patients (CP, *n* = 10), and patients with invasive aspergillosis (IA, *n* = 25) or mucormycosis (MU, *n* = 7) was analysed by flow cytometry. Samples containing fewer than 100 viable lymphocytes (CD45^+^ cells) were excluded (a detailed gating strategy is provided in Figure S1). Individual percentages are depicted with mean (bar) ± standard deviation (red). Significant differences between all groups were calculated using a nonparametric Kruskal‐Wallis test followed by Dunn’s post hoc test and are indicated by asterisks (*p ≤ 0.05, **p ≤ 0.01).

**Table S1.** Detailed list of antibodies. Detailed list of antibodies used for flow cytometry.

**Table S2.** Patient characteristics.

## Data Availability

The data that support the findings of this study are available from the corresponding author upon reasonable request.
