# Supplementary figures and images for: Immune Phenotypes in Patients With Invasive Mould Infection Support the Use of PD‐1 Inhibition as Potential Treatment Option
Source: Mycoses. 2025 Mar 17;68(3):e70044. doi: 10.1111/myc.70044 (PMC11912816; doi:10.1111/myc.70044)

Supplementary Figure 1

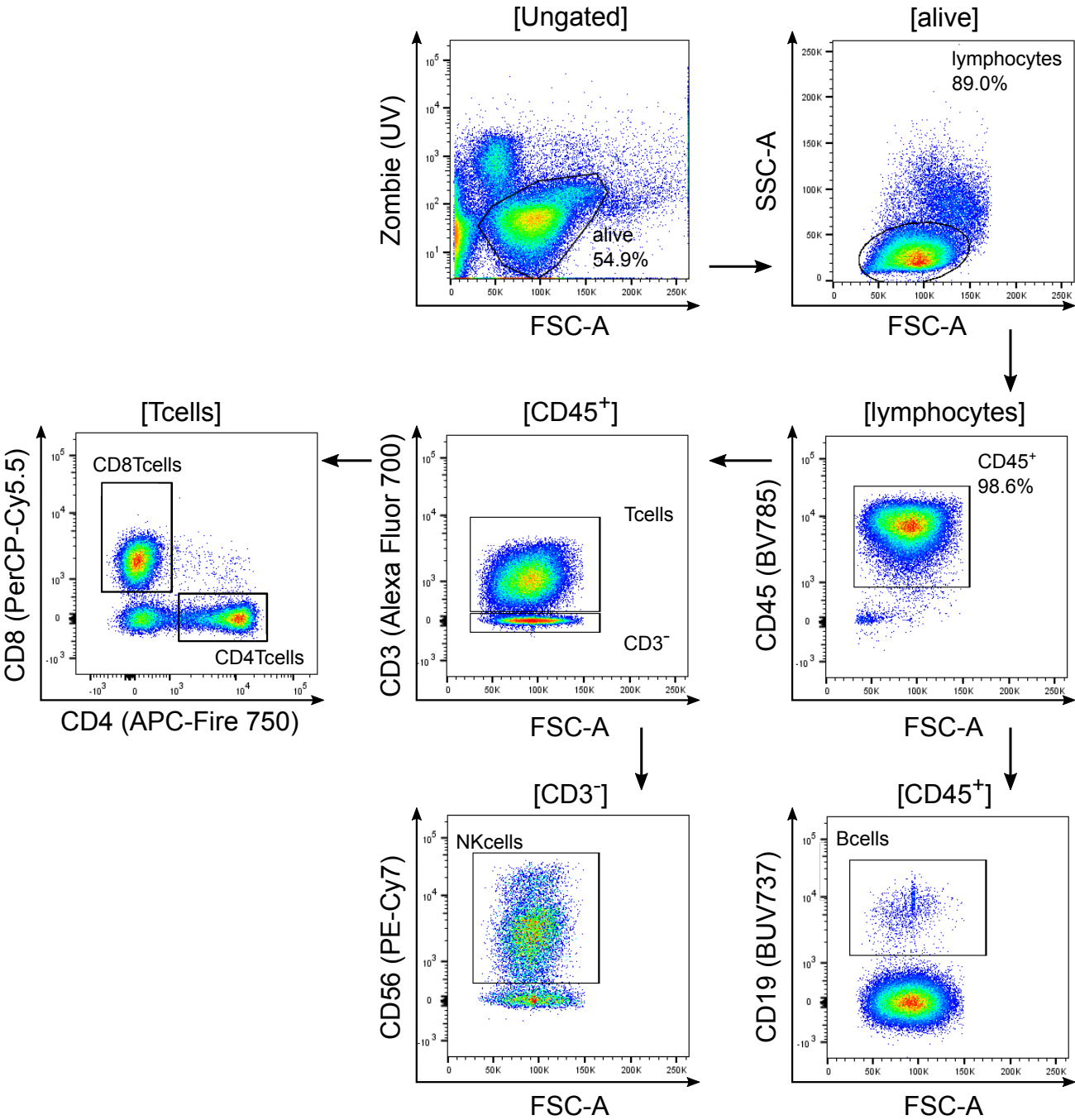

Supplement: Supplementary file 1 — Figure S1. Gating strategy. Peripheral blood mononuclear cells were analysed by flow cytometry. Dead cells were excluded (alive). Lymphocytes were selected by gating for size and granularity (forward scatter (FCS‐A) and side scatter (SSC‐A)). Living lymphocytes were further gated for CD45+ lymphocytes (CD45+). B cells were defined by gating on CD19, whereas T cells were defined as CD3+ cells. T cells were then gated for their expression of CD4 and CD8. CD3 negative cells expressing CD56 were considered NK cells. A detailed list of antibodies, clones, and fluorochromes is provided in Table S1. [file MYC-68-e70044-s006.pdf]

Supplementary Figure 2

A

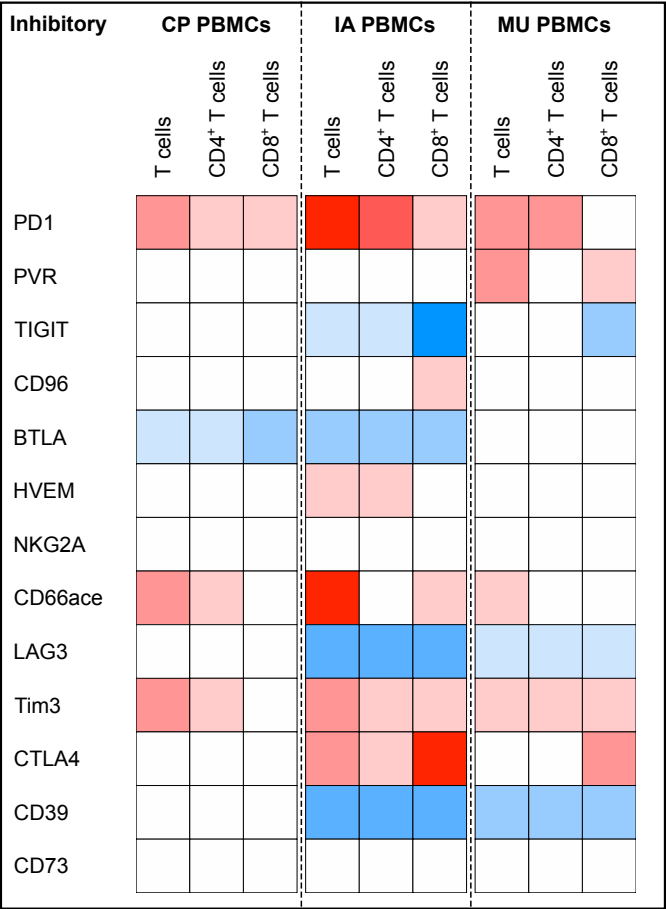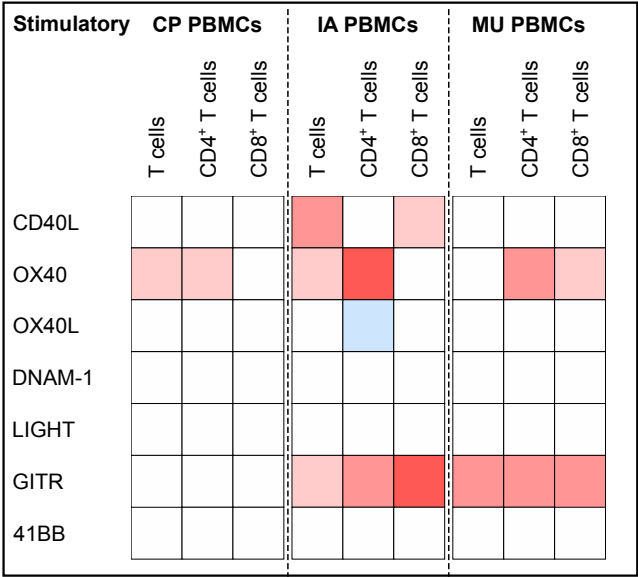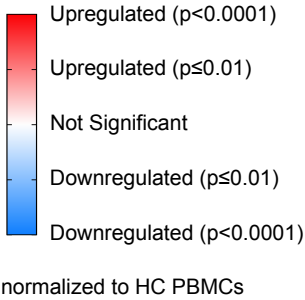

Supplement: Supplementary file 2 — Figure S2. Significant alteration of immune checkpoint molecule expression in patient PBMCs. The expression of immune checkpoint molecules on T cells, CD4+ T cells, and CD8+ T cells in PBMCs from healthy controls (HC, n = 5), cancer patients (CP, n = 10), and patients with invasive aspergillosis (IA, n = 25) or mucormycosis (MU, n = 7) was analysed by flow cytometry. Samples containing fewer than 100 viable lymphocytes (CD45+ cells) were excluded (a detailed gating strategy is provided in Figure S1). Significant differences of immune checkpoint molecule expression between HCs and CP, IA, or MU PBMCs were calculated using a tow‐tailed, nonparametric Mann–Whitney test. [file MYC-68-e70044-s003.pdf]

Supplementary Figure 3

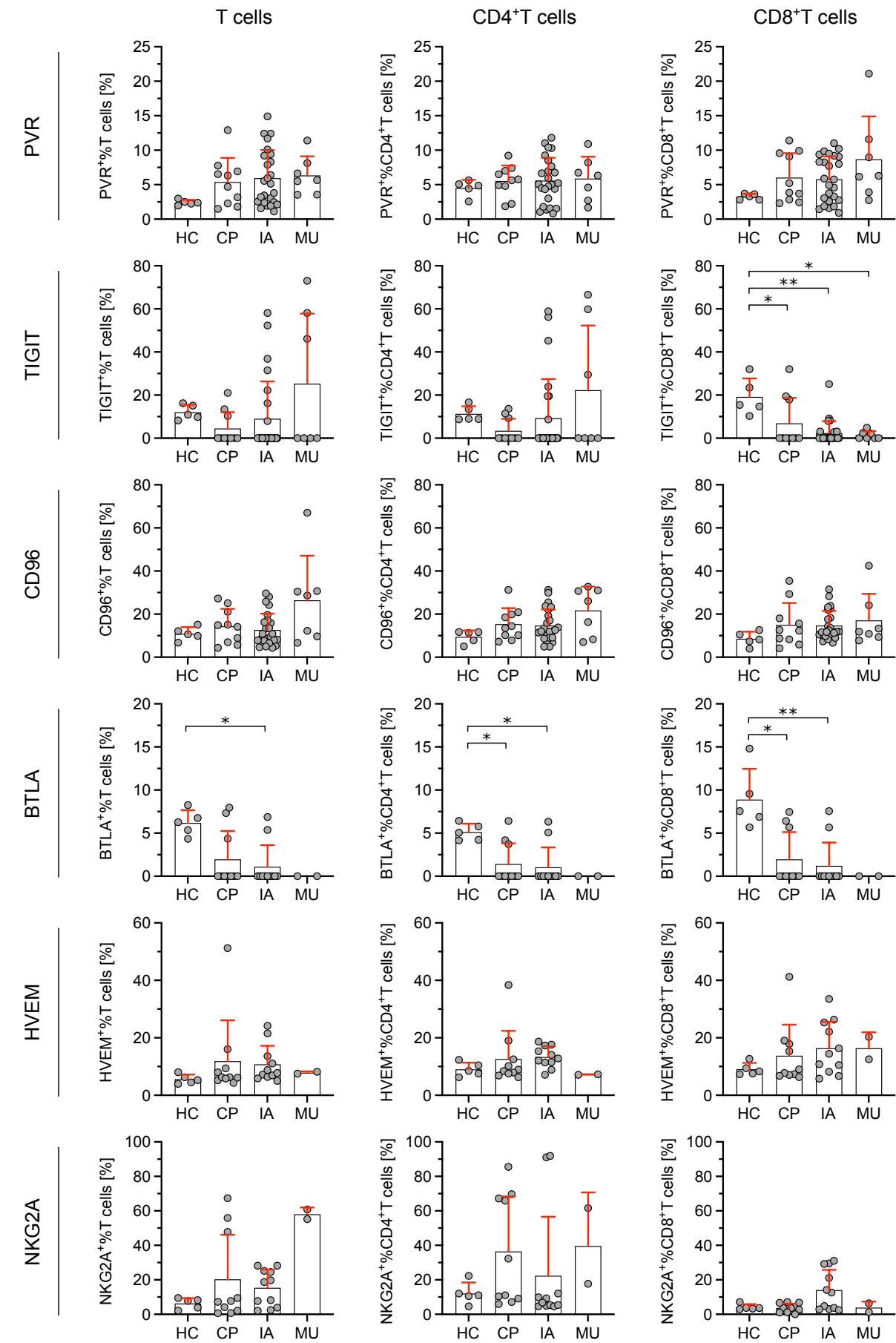

Supplement: Supplementary file 3 — Figure S3. Co‐inhibitory molecule expression in patient PBMCs. The expression of co‐inhibitory molecules on T cells, CD4+ T cells, and CD8+ T cells in PBMCs from healthy controls (HC, n = 5), cancer patients (CP, n = 10), and patients with invasive aspergillosis (IA, n = 25) or mucormycosis (MU, n = 7) was analysed by flow cytometry. Samples containing fewer than 100 viable lymphocytes (CD45+ cells) were excluded (a detailed gating strategy is provided in Figure S1). Individual percentages are depicted with mean (bar) ± standard deviation (red). Significant differences between all groups were calculated using a nonparametric Kruskal‐Wallis test followed by Dunn’s post hoc test and are indicated by asterisks (*p ≤ 0.05, **p ≤ 0.01). [file MYC-68-e70044-s004.pdf]

Supplementary Figure 4

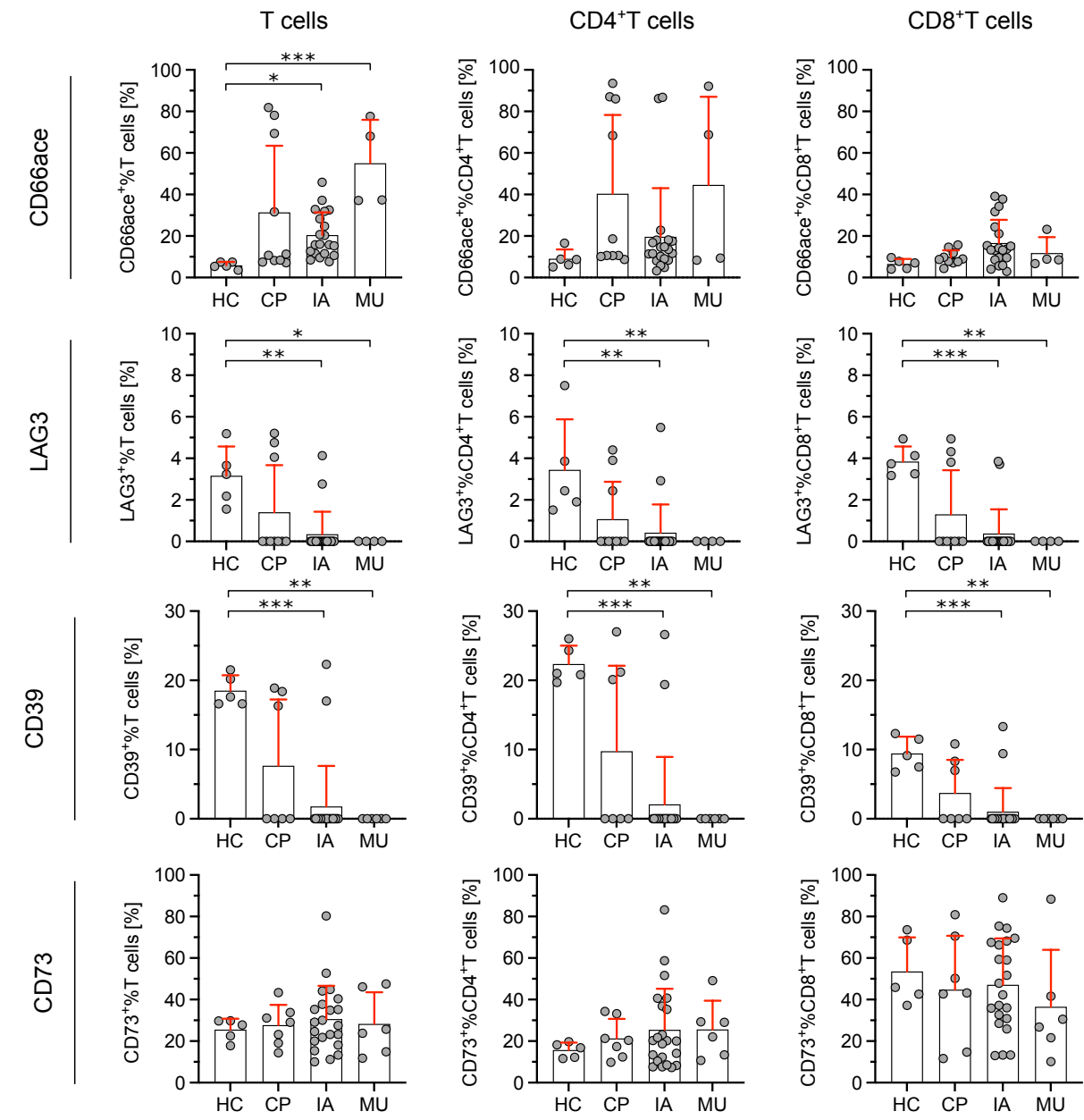

Supplement: Supplementary file 4 — Figure S4. Co‐inhibitory molecule expression in patient PBMCs. The expression of co‐inhibitory molecules on T cells, CD4+ T cells, and CD8+ T cells in PBMCs from healthy controls (HC, n = 5), cancer patients (CP, n = 10), and patients with invasive aspergillosis (IA, n = 25) or mucormycosis (MU, n = 7) was analysed by flow cytometry. Samples containing fewer than 100 viable lymphocytes (CD45+ cells) were excluded (a detailed gating strategy is provided in Figure S1). Individual percentages are depicted with mean (bar) ± standard deviation (red). Significant differences between all groups were calculated using a nonparametric Kruskal‐Wallis test followed by Dunn’s post hoc test and are indicated by asterisks (*p ≤ 0.05, **p ≤ 0.01, ***p ≤ 0.001). [file MYC-68-e70044-s002.pdf]

## Supplementary Figure 5

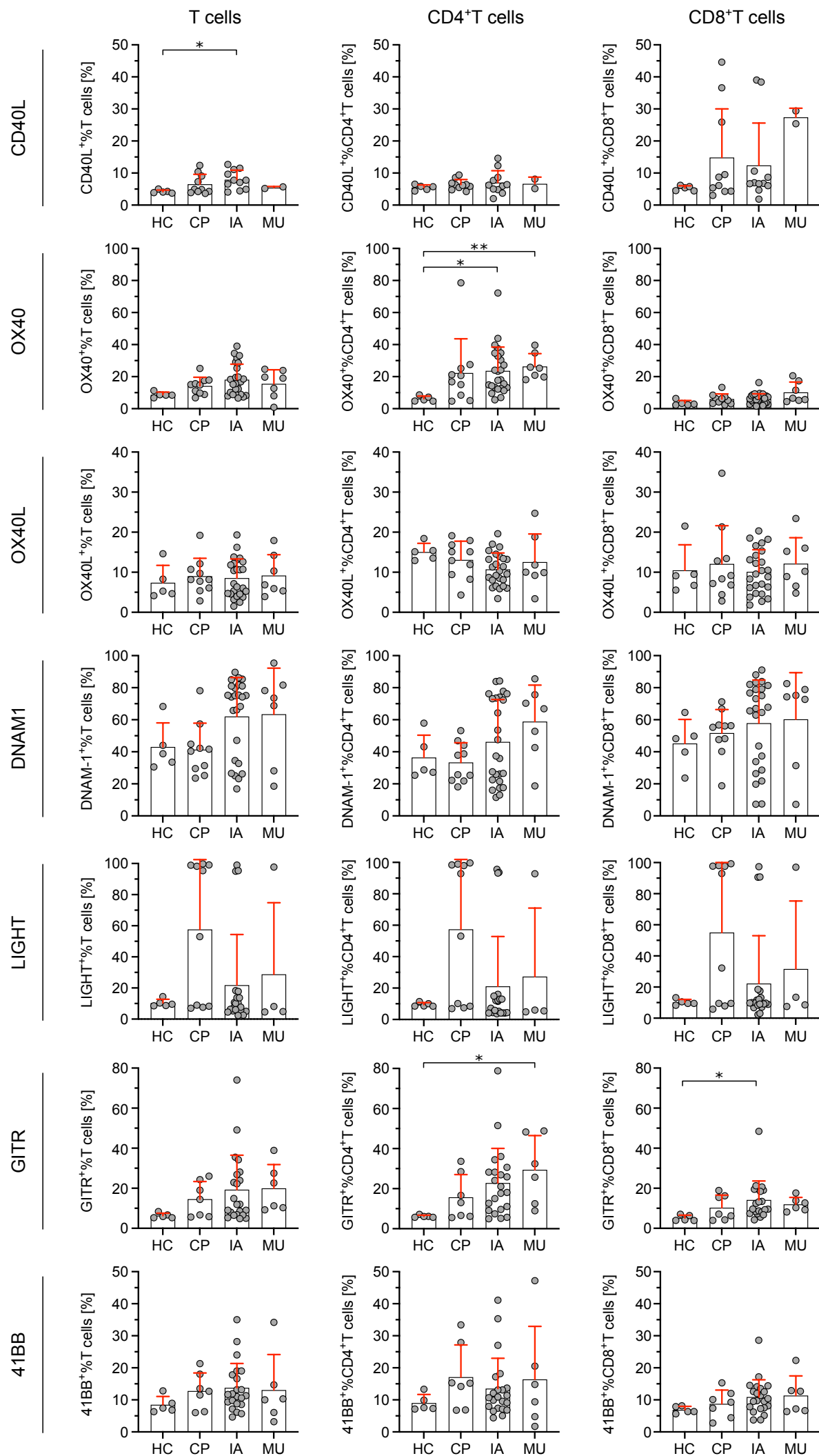

Supplement: Supplementary file 5 — Figure S5. Co‐stimulatory molecule expression in patient PBMCs. The expression of co‐stimulatory molecules on T cells, CD4+ T cells, and CD8+ T cells in PBMCs from healthy controls (HC, n = 5), cancer patients (CP, n = 10), and patients with invasive aspergillosis (IA, n = 25) or mucormycosis (MU, n = 7) was analysed by flow cytometry. Samples containing fewer than 100 viable lymphocytes (CD45+ cells) were excluded (a detailed gating strategy is provided in Figure S1). Individual percentages are depicted with mean (bar) ± standard deviation (red). Significant differences between all groups were calculated using a nonparametric Kruskal‐Wallis test followed by Dunn’s post hoc test and are indicated by asterisks (*p ≤ 0.05, **p ≤ 0.01). [file MYC-68-e70044-s001.pdf]
