# Supplementary material for: Immune Phenotypes in Patients With Invasive Mould Infection Support the Use of PD‐1 Inhibition as Potential Treatment Option
Source: Mycoses. 2025 Mar 17;68(3):e70044. doi: 10.1111/myc.70044 (PMC11912816; doi:10.1111/myc.70044)
Supplement: Supplementary file 6 — Table S1. Detailed list of antibodies. Detailed list of antibodies used for flow cytometry. [file MYC-68-e70044-s007.pdf]

Supplementary Table 1

Antibody	Fluorochrome	Clone	Origin	Company	Catalogue
CD8a	FITC	HIT8a	mouse	Biolegend	300906
CD20	FITC	2H7	mouse	Biolegend	302304
CD8	PerCP/Cy5.5	SK1	mouse	Biolegend	344710
CD357 (GITR)	PerCP/Cy5.5	108-17	mouse	Biolegend	371218
OX40L	PE	11C3.1	mouse	Biolegend	326308
CD96 (TACTILE)	PE	NK92.39	mouse	Biolegend	338406
CD270 (HVEM)	PE	122	mouse	Biolegend	318806
CD258 (LIGHT)	PE	T5-39	mouse	Biolegend	318706
CD152 (CTLA4)	PE	BNI3	mouse	BD	555853
CD134 (OX40)	PE-Dazzle 594	Ber-ACT35	mouse	Biolegend	350020
CD155 (PVR)	PE-Dazzle 594	SKII.4	mouse	Biolegend	337616
CD154 (CD40L)	PE-Dazzle 594	24-31	mouse	Biolegend	310840
CD366 (Tim3)	PE-Dazzle 594	F38-2E2	mouse	Biolegend	345034
CD56	PE-Cy7	5.1H11	mouse	Biolegend	362510
CD73	PE-Cy7	A2D	mouse	Biolegend	344010
PD1	Alex Fluor 647	EH12.1	mouse	BD	560838
CD226 (DNAM-1)	APC	11A8	mouse	Biolegend	338312
NKG2A	APC	REA110	human cell line	Miltenyi	130-113-563
CD66ace	APC	ASL-32	mouse	Biolegend	342308
FoxP3	Alexa Fluor 647	259D	mouse	Biolegend	320214
CD3	Alexa Fluor 700	SK7	mouse	Biolegend	344822
CD4	APC-Fire 750	SK3	mouse	Biolegend	344638
PD-L1	BV421	29E.2A3	mouse	Biolegend	329714
TIGIT	BV421	A15153G	mouse	Biolegend	372710
CD272 (BTLA)	BV421	MIH26	mouse	Biolegend	344512
CD223 (LAG3)	BV421	11C3C65	mouse	Biolegend	369314
CD357 (GITR)	BV421	108-17	mouse	Biolegend	371208
CD39	BV421	A1	mouse	Biolegend	328214
CD137 (4-1BB)	BV421	4B4-1	mouse	Biolegend	309820
CD154 (CD40L)	BV605	24-31	mouse	Biolegend	310826
CD137 (4-1BB)	BV605	4B4-1	mouse	Biolegend	309822
CD45	BV785	HI30	mouse	Biolegend	304048
CD19	BUV737	SJ25C1	mouse	BD	564303
Zombie	UV			Biolegend	423108
