# Supplementary material for: Immune Phenotypes in Patients With Invasive Mould Infection Support the Use of PD‐1 Inhibition as Potential Treatment Option
Source: Mycoses. 2025 Mar 17;68(3):e70044. doi: 10.1111/myc.70044 (PMC11912816; doi:10.1111/myc.70044)
Supplement: Supplementary file 7 — Table S2. Patient characteristics. [file MYC-68-e70044-s005.pdf]

Supplementary Table 2 Patient characteristics

Characteristic	IA patients<br>n [% of n=25]	MU patients<br>n [% of n=7]	CP patients<br>n [% of n=10]	HC<br>n [% of n=5]
**Sex**				
Female	11 [44.0]	0 [0.0]	6 [60.0]	2 [40.0]
Male	14 [56.0]	5 [100.0]	4 [40.0]	3 [60.0]
**Age** in years (mean [range])	53 [18-79 years]	51 [15-71 years]	48 [22 – 64 years]	47 [26 – 59 years]
**Risk factors**				
Haematological underlying disease				
Acute leukaemia	15 [60.0]	5 [100.0]	5 [50.0]	
MDS	2 [8.0]	0 [0.0]	1 [10.0]	
Aggressive lymphoma	3 [12.0]	0 [0.0]	3 [30.0]	
Multiple Myeloma	1 [4.0]	0 [0.0]	0 [0.0]	
Oncological underlying disease				
Melanoma	1 [4.0]	0 [0.0]	0 [0.0]	
Immunosuppression				
Chemoimmunotherapy	22 [88.0]	5 [100.0]	0 [0.0]	
Neutropenia	17 [68.0]	4 [80.0]	3 [60.0]	
Viral pneumonia (influenza/COVID-19)	2 [8.0]	0 [0.0]	1 [10.0]	
Allogeneic haematopoietic stem-cell transplantation	3 [12.0]	3 [60.0]	2 [20.0]	
None	1 [4.0]	0 [0.0]	4 [40.0]	
**Classification of invasive fungal infection along EORTC**				
Probable	19 [76.0]	0 [0.0]		
Proven	6 [24.0]	5 [100.0]		
**Outcome**				
Survival d30	15 [60.0]	1 [20.0]	10 [100.0]	
Survival d90	14 [56.0]	1 [20.0]	8 [80.0]	
